# Fibrinolytic markers could be useful predictors of severity in patients with pulmonary arterial hypertension: a retrospective study

**DOI:** 10.1186/s12959-021-00332-4

**Published:** 2021-11-04

**Authors:** Makoto Shoji, Taiju Matsui, Hideaki Tanaka, Kosuke Nomura, Hiroaki Tsujita, Yusuke Kodama, Shinji Koba, Youichi Kobayashi, Toshiro Shinke

**Affiliations:** Department of Medicine, Division of Cardiology, Showa University School of Medicine, 1-5-8 Hatanodai, Shinagawa-ku, Tokyo, 142-8666 Japan

**Keywords:** tPAI-1, Thrombomodulin, Pulmonary artery hypertension

## Abstract

**Background:**

The severity of pulmonary arterial hypertension (PAH) is classified based on mean pulmonary artery pressure (mPAP) levels. However, other markers have not been elucidated. Fibrinolytic markers, such as total plasminogen activator inhibitor-1 (tPAI-1) and thrombomodulin (TM), are known to reflect arterial endothelial function. However, the relationship between serum tPAI-1, TM and pulmonary circulation has not been completely determined.

**Methods:**

This study included 100 consecutive patients (38 men), with a mean age of 68.9 ± 12.0 years, with cardiac diseases who underwent right heart catheterization. Serum coagulation and fibrinolytic marker levels were measured.

**Results:**

The average mPA*P* value was 25.1 ± 13.1 mmHg for all patients. The mPAP levels revealed a significant positive correlation with serum tPAI-1 (*ρ* = 0.24, *p* = 0.042) and uric acid (*ρ* = 0.29, *p* = 0.0031) levels. In the group with mPAP levels less than 25 mmHg (*n* = 58, ave. 17.3 ± 4.3 mmHg), mPAP levels showed a significant positive correlation with serum tPA-1 (*ρ* = 0.34, *p* = 0.034) and TM (*ρ* = 0.34, *p* = 0.043) values. The mean tPAI-1 (29.8 ± 23.3 ng/ml, *p* = 0.047) and uric acid (5.7 ± 1.8 mg/dl, *p* = 0.026) levels were significantly less in those with lower mPAP levels. A multivariate analysis revealed that tPAI-1 alone was a significant independent characteristic marker of PAH (odds ratio 1.02, 95%CI 1.000–1.036, *p* = 0.034).

**Conclusions:**

These results indicate that serum tPAI-1 and TM may be useful predictors of severity, similar to mPAP in patients with PAH. They could be beneficial in predicting PAH among patients in the early stage of the disease.

## Background

The severity of pulmonary artery hypertension (PAH) is classified based on the mean pulmonary artery pressure (mPAP) levels that are evaluated by right heart catheterization (RHC). It is a fetal disease that is reported to have a poor prognosis despite the development in medication [1, 2]. The mPAP has been established as an accurate prognostic marker for PAH in previous reports [3, 4]. However, the patients require RHC to evaluate mPAP, which is an invasive procedure. Thus, non-invasive and alternative predictors for prognosis are needed.

The fibrinolytic marker, total plasminogen activator inhibitor-1 (tPAI-1) is a major inhibitor of plasminogen activator in plasma. It is stored in platelets and has several sources, including the vascular endothelium [5]. tPAI-1 is known to be associated with a proinflammatory atherosclerotic risk. Moreover, there are reports that tPAI-1 is independently associated with the degree of intima media thickness (IMT) [6, 7]. Thrombomodulin (TM) has an anticoagulant effect that is activating protein C, and this is corroborated by reports about elevated blood levels of TM in patients with acute pulmonary embolism [8, 9]. It is also known that TM is metabolized by the liver and excreted through the kidneys, and its level is elevated in a variety of diseases [10].

These fibrinolytic markers, tPAI-1 and TM, are known to reflect arterial endothelial function. However, the relationship between serum tPAI-1, TM and pulmonary circulation has not been completely elucidated. In the present study, we aimed to evaluate if tPAI-1 and TM could be used as predictors of severity in patients with PAH.

## Methods

We retrospectively reviewed the medical records of all consecutive patients who underwent RHC to assess cardiac function in Showa University Hospital between January 1, 2012, and March 31, 2018. This study was approved by the ethics committee of Showa University School of Medicine.

The details about patient history, physical examination, blood investigations, and echocardiography were evaluated. Patients with the following diseases that could affect serum tPAI-1 and TM levels were excluded from the study: (1) those on hemodialysis [11]; (2) those currently under treatment for malignant neoplasms; (3) those with hepatic failure [12, 13]; (4) those with congestive heart failure (CHF) NYHA grade III or more.

Blood samples were collected from patients at admission before RHC was performed. Fibrinolytic and coagulation marker levels, including tPAI-1, TM, D-dimer, thrombin-antithrombin complex (TAT), plasmin-α2 plasmin inhibitor complex (PIC), and prothrombin fragment 1 + 2 (PTF 1 + 2) were also measured. The tPAI-1 level was measured using the latex agglutination test (JCA-BM9130, JEOL Ltd., Tokyo, Japan). The TM level was measured using an enzyme-linked immunoassay (ELISA) kit, AP-X (Kyowa Medex Co Ltd., Tokyo, Japan) [14].

### Statistical analysis

Data are reported as mean ± standard deviation. Continuous and categorical variables were compared using the Mann-Whitney *U* test or chi-squared test, as appropriate. We performed Cox’s stepwise logistic regression analysis to identify significant independent characteristic markers that were related to mPAP. We then calculated the odds ratios (ORs) and 95% confidence intervals (CIs). Correlation analysis was performed using Spearman’s rank correlation. We considered *p* values less than 0.05 to be statistically significant. JMP software version 14.0 (SAS, Cary, NC, USA) was used for the statistical analysis.

## Results

We reviewed the medical records of 111 consecutive patients who underwent RHC. Of these, 4 were excluded because of ongoing hemodialysis, 3 for currently undergoing treatment of a malignant neoplasm, 2 for hepatic failure, and 2 for worsening CHF (NYHA grade III or more). The remaining 100 patients were included in the study, and their clinical characteristics are shown in Tables 1, 2.

**Table 1:** Patient characteristics

	Total Patients
N	100
Baseline characteristics	
Age (years)	68.9 ± 12.0
Male	38 (38%)
BMI	21.8 ± 4.2
Hypertension	42 (42%)
Diabetes mellitus	11 (11%)
Heart failure	30 (30%)
Collagen diseases	21 (21%)
Valvular diseases	11 (11%)
Pulmonary embolism	11 (11%)
Congenital heart diseases	9 (9%)
Respiratory diseases	7 (7%)
Ischemic heart diseases	6 (6%)
Idiopathic pulmonary hypertension	5 (5%)
Taking anticoagulants	41 (41%)
Hemodynamics	
Mean pulmonary artery pressure (mmHg)	25.1 ± 13.2
Pulmonary capillary wedge pressure (mmHg)	13.4 ± 9.0
Right ventricular pressure (mmHg)	40.7 ± 20.4
Echocardiography	
Ejection Fraction (%)	56.3 ± 12.8
Right Ventricular systolic pressure (mmHg)	51.2 ± 21.5
BNP (pg/ml)	464.8 ± 1367
Uric Acid (mg/dl)	6.1 ± 1.9
Estimated GFR (ml/min)	61.4 ± 21.8

Data are presented as mean ± SD or n (%)

*BMI* Body mass index

**Table 2:** Coagulation and Fibrinolytic markers

Makers	
D-dimer (μg/ml)	3.24 ± 11.5
Thrombomodulin (FU/ml)	3.0 ± 1.3
tPAI-1 (ng/ml)	38.0 ± 39.8
TAT (μg/l)	6.33 ± 11.1
PIC (μg/ml)	1.5 ± 2.8
PT F1 + 2 (pmol/l)	258 ± 217

Data are presented as mean ± SD or n (%)

*tPAI-1* Total plasminogen activator inhibitor-1; *TAT* Thrombin antithrombin complex, *PIC* plasmin-α plasmin inhibitor complex, *PT F1 + 2* Prothrombin-fragment 1 + 2

The mean age of the participants was 68.9 ± 12.0 years, and thirty-eight patients (38%) were men. The underlying conditions comprised heart failure in 30 patients (30%), collagen diseases in 21 (21%), valvular diseases in 11 (11%), pulmonary embolism in 11 (11%), congenital heart diseases in 9 (9%), respiratory diseases in 7 (7%), ischemic heart diseases in 6 (6%), and idiopathic pulmonary hypertension in 5 (5%) (Table 1).

**Fig. 1:**
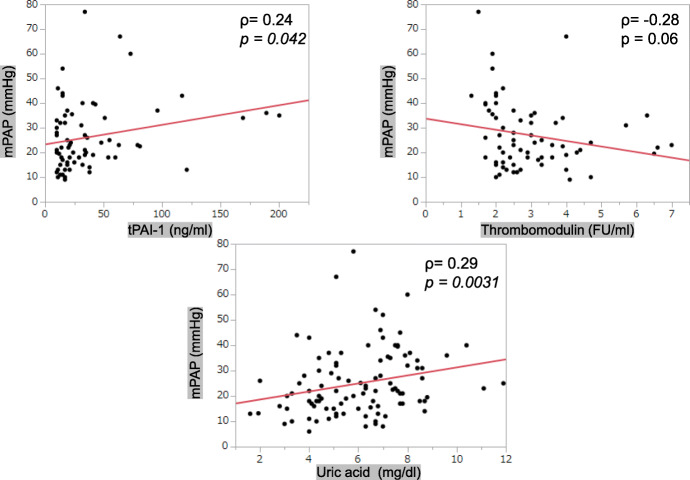
Relationship between the mPAP levels and serum markers. A significant positive correlation is noted between the mPAP levels with serum tPAI-1 and uric acid values

**Fig. 2:**
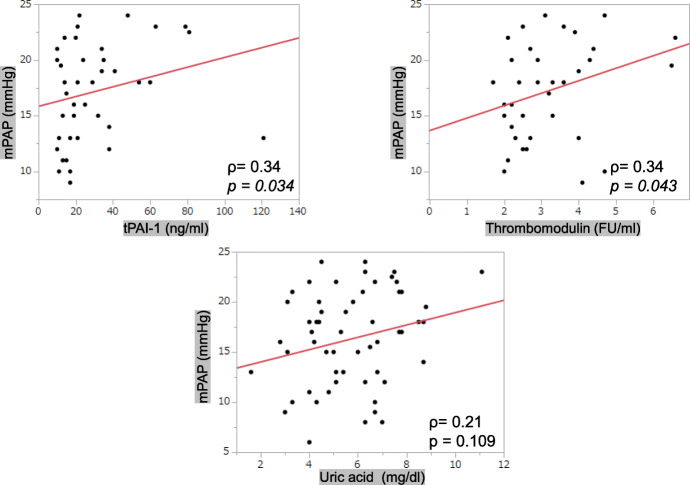
Relationship between the mPAP and serum markers in those with levels less than 25 mmHg. The mPAP levels had significant positive correlation with serum tPAI-1 and TM values

The average mPAP value was 25.1 ± 13.1 mmHg. The mPAP values had significant positive correlation with serum tPAI-1 (*ρ* = 0.24, *p* = 0.042) and uric acid (*ρ* = 0.29, *p* = 0.0031) levels (Table 1, Fig. 1). After categorizing the patients based on their mPAP values, those in the group with average mPAP values less than 25 mmHg (17.3 ± 4.3 mmHg) were observed to have a significant positive correlation between serum tPA-1 (*ρ* = 0.34, *p* = 0.034) and TM (*ρ* = 0.34, *p* = 0.043) levels and mPAP values (Fig. 2).

Both mean tPAI-1 (29.8 ± 23.3 ng/ml, *p* = 0.047) and uric acid (5.7 ± 1.8 mg/dl, *p* = 0.026) levels were significantly less in the group with lower mPAP values. A multivariate analysis, adjusted for age, gender, and BMI, revealed that tPAI-1 level alone was an independent characteristic marker (odds ratio 1.02, 95%CI 1.000–1.036, *p* = 0.034) of the severity of PAH (Tables 3, 4).

**Table 3:** Comparison of biomarkers, mPAP< 25 or not

	mPAP< 25	mPAP≥25	*P* value
N	58	42	
D-dimer	2.0 ± 2.4	4.9 ± 18	N.S.
Thrombomodulin	3.3 ± 1.3	2.6 ± 1.1	N.S.
tPAI-1	29.8 ± 23.3	49.0 ± 52.5	0.047
TAT	5.0 ± 9.2	8.1 ± 12.9	N.S.
PIC	1.2 ± 0.7	1.9 ± 4.1	N.S.
PT F1 + 2	262.7 ± 171.1	252.7 ± 260.7	N.S.
BNP	249.2 ± 294.9	752.2 ± 2024	N.S.
Uric Acid	5.7 ± 1.8	6.6 ± 2.0	0.026
Estimated GFR	63.9 ± 22.5	58.1 ± 20.4	N.S.

**Table 4:** Multivariate logistic regression adjusted to age, gender, BMI

	Multivariate logistic regression	
	OR (95% CI)	*P* value
tPAI-1	1.02 (1.000–1.036)	0.034
UA	1.07 (0.793–1.434)	N.S.

Multivariate logistic regression adjusted to age, gender, BMI

*OR* Odds ratio, *CI* Confidence interval

## Discussion

The study results indicate that mPAP values have a significant positive correlation with serum tPAI-1 and uric acid levels in all enrolled patients. Among patients with mPAP values less than 25 mmHg PAP, a significant positive correlation is observed with serum tPAI-1 and TM levels. The uric acid levels revealed a lower degree of association than the tPAI-1 levels, which was found to be an independent marker in the group with lower mPAP values.

The severity of PAH in patients is indicated by the mPAP levels evaluated by RHC, which is an invasive examination. Therefore, less-invasive and reliable biomarkers that reflect the disease status accurately are desired. Serum uric acid is a traditional biomarker that reflects endothelial function and is a potential risk factor for arteriosclerotic change [15, 16]. In the present study, uric acid levels revealed a significant positive correlation with mPAP values in all enrolled patients. This result is supported by the findings in some previous studies on atherosclerosis. However, in the group with mPAP values less than 25 mmHg, uric acid levels did not show a significant association with mPAP.

According to the diagnostic criteria for PAH, an mPAP level of 25 mmHg is the cut-off value. Many clinicians focus on the preclinical stage of PAH predominantly, and the patients with mPAP levels less than 25 mmHg should be on intensive, careful follow-up because the response to treatment changes dramatically according to the state of the disease [1–4]. Hence, we focused our investigations in this group. The results indicate that unlike serum uric acid levels, tPAI-1 levels alone are an independent characteristic marker in the group with lower mPAP levels. Additionally, the mPAP levels reveal a significant positive association with serum tPAI-1 and TM but not uric acid levels, which means that these markers could be potential predictors of severity in patients with PAH in their early stages.

tPAI-1 is an inhibitor of plasminogen, which regulates the systemic fibrinolytic system, and the levels indicate microvascular injury due to various conditions, including diabetes mellitus [17]. It complements the activities of protein anticoagulants and endothelial-derived platelet inhibitors, such as proteins C, S, anti-thrombin III, and nitric oxide [5]. It is an endogenous defense mechanism for the prevention of atherosclerotic change, and there are some reports that the tPAI-1 levels are positively and independently associated with the degree of IMT [6, 7].

In contrast, TM is a factor that regulates the anticoagulation system by capturing thrombin and activating protein C. Once damage to endothelial cells occurs, TM expressed on endothelial cells is cleaved from the membrane, and the product is detectable in a soluble form in circulating peripheral blood. This indicates that TM can be a marker of endothelial cell damage [18–20]. The mechanism in advanced PAH is speculated to involve an increased release of tPAI-1 and TM from smooth muscle and endothelial cells caused by pulmonary vascular damage. This could result in elevated serum tPAI-1 and TM levels, as indicated in the present study. Ogawa et al. suggested that thrombin modulates pulmonary circulation through activation of the Akt pathway in smooth muscle cells among patients with PAH and chronic thromboembolic pulmonary hypertension (CTEPH) [21]. It also meant that TM levels could be altered due to endothelial dysfunction in the pulmonary artery. However, the concentration of circulating serum TM in peripheral blood would be saturated and the rest would be recruited by the kidney. In fact, there were no significant differences in the concentration of TM between the groups with mPAP levels less than or more than 25 mmHg.

We have demonstrated that mPAP levels have a significant positive correlation with serum tPAI-1 and TM but not uric acid values, which means that these markers could be potential predictors of severity in patients with PAH, especially in their early stages with mPAP levels less than 25 mmHg.

This study has few limitations. First, this study was retrospective in nature with its inherent defects; therefore, a prospective study is needed in the future to confirm the results. Second, the population size is small, and the patients were from heterogeneous backgrounds. A larger sample size that includes a larger cohort of severe cases would allow a more reliable statistical evaluation. Third, not plasma but serum was used to evaluate tPAI-1 and other markers in this study based on previous study [14]. Serum is the supernatant of clotted blood where platelets have released their contents. Hence there is a possibility that the value of tPAI-1 is overestimated since it is a component of the platelet releasate.

## Conclusion

These results reveal that serum tPAI-1 and TM may be useful predictors that are similar to mPAP in patients with PAH. They could be beneficial in predicting severity in patients with PAH, especially in their early stages.

## Abbreviations

BMI: Body mass index
CHF: Congestive heart failure
CI: Confidence intervals
CTEPH: Chronic thromboembolic pulmonary hypertension
ELISA: Enzyme-linked immune sorbent assay
IMT: Intima media thickness
OR: Odds ratio
PIC: Plasmin-α2 plasmin inhibitor complex
PT F1 + 2: Prothrombin-fragment 1 + 2
RHC: Right heart catheterization
SD: Standard deviation
TAT: Thrombin antithrombin
tPAI-1: Total plasminogen activator inhibitor-1
TM: Thrombomodulin
